# Contribution of Copper Slag to Water Treatment and Hydrogen Production by Photocatalytic Mechanisms in Aqueous Solutions: A Mini Review

**DOI:** 10.3390/ma17225434

**Published:** 2024-11-07

**Authors:** Susana I. Leiva-Guajardo, Norman Toro, Edward Fuentealba, Mauricio J. Morel, Álvaro Soliz, Carlos Portillo, Felipe M. Galleguillos Madrid

**Affiliations:** 1 Centro de Desarrollo Energético Antofagasta, Universidad de Antofagasta, Antofagasta 1240000, Chile; edward.fuentealba@uantof.cl (E.F.); carlos.portillo@uantof.cl (C.P.); 2 Facultad de Ingeniería y Arquitectura, Universidad Arturo Prat, Iquique 1100000, Chile; notoro@unap.cl; 3 Departamento de Química y Biología, Universidad de Atacama, Av. Copayapú 485, Copiapó 1530000, Chile; mauricio.morel@uda.cl; 4 Departamento de Ingeniería en Metalurgia, Universidad de Atacama, Av. Copayapú 485, Copiapó 1530000, Chile; alvaro.soliz@uda.cl

**Keywords:** solar hydrogen, waste material, copper slag, photocatalysis, water treatment

## Abstract

Hydrogen has emerged as a promising energy carrier, offering a viable solution to meet our current global energy demands. Solar energy is recognised as a primary source of renewable power, capable of producing hydrogen using solar cells. The pursuit of efficient, durable, and cost-effective photocatalysts is essential for the advancement of solar-driven hydrogen generation. Copper slag, a by-product of copper smelting and refining processes, primarily consists of metal oxides such as hematite, silica, and alumina. This composition makes it an attractive secondary resource for use as a photocatalyst, thereby diverting copper slag from landfills and generating 0.113 μmol/g h of hydrogen, as noted by Montoya. This review aims to thoroughly examine copper slag as a photocatalytic material, exploring its chemical, physical, photocatalytic, and electrochemical properties. Additionally, it evaluates its suitability for water treatment and its potential as an emerging material for large-scale solar hydrogen production.

## 1. Introduction

The rapid expansion of the global population and social progress have resulted in a continuous increase in the demand for energy and fresh water. This growing need, largely driven by the consumption of fossil fuels, is profoundly impacting our atmosphere and contributing to a concerning rise in the Earth’s average temperature [1]. The crucial task of decarbonising our energy systems is essential to achieving the goals set in 2015. This international agreement, ratified by 196 nations and enacted in 2016, commits to limiting global warming to no more than 2 °C above pre-industrial levels [2]. Additionally, it established the need to reduce greenhouse gas emissions by close to 70% in 2050 [3]. Hydrogen (H_2_) gas is the new energy vector [4]. H_2_ is a non-toxic fuel, notable for its exceptional clean-burning properties, capable of producing up to 120 MJ/kg under standard conditions. This output significantly surpasses the energy yield of any fossil fuel [5].

Copper Slag (CS) is a residue produced in a pyrometallurgical process to obtain copper concentrated from a concentrate of sulfide minerals and materials like FeO, SiO_2_, Al_2_O_3_, CaO, and Cu, respectively [6]. Now, it does not have very good economic value in the market. CS has been used in the pavement industry, bituminous pavements, concrete, blended cement, abrasive tools, cutting tools, tiles and glass, roofing granules, and asphalt concrete aggregate [6,7,8,9]. CS has always been considered as a construction material; however, Huanosta-Gutiérrez et al., in 2012, suggested the possible use of CS as an electrocatalytic material for the depletion of pollutants present in the water [10], and Montoya et al. used CS as an economically and viable material to eliminate contaminants in the water, reduce the impact of industry water waste, and generate 0.113 μmol/g h of H_2_ [11] from toxic alcohols by photocatalytic degradation [12,13].

CS is one of the main solid wastes from the pyrometallurgical process applied to the concentrates in the copper industry plants. Chile produces approximately 4.5 million tons of slag per year, and more than 30 million tons of CS are produced annually in the world, which demonstrates the magnitude of the waste from the processing of mining ores. In addition, CS often contains a large amount of valuable metal and is now considered a secondary metal resource [14,15]. These metals could be Fe, Cr, Cu, Al, Zn, Co, Ni, Nb, Ta, and Au and can be recovered by operating units considered in slag ore processing, such as (i) crushing, (ii) grinding, (iii) magnetic separation, (iv) flotation, (v) leaching, and (vi) roasting. Therefore, the recycling of metals present in CS can not only recover strategic metals but also contribute to carbon neutrality [16,17]. In terms of appearance and properties, naturally cooled CS has a black colour and a glassy surface, and it is dense, lumpy, hard, and brittle. However, depending on the cooling pattern, which can be natural or water-quenched, CS can differ in appearance, density, and shape [18]. In recent years (1999–2019), approximately 752 Mt of CS has been generated worldwide, where the largest generation of this waste occurs in South America (40%), followed by Asia (13%), USA (8%), Oceania (6%), Europe (6%), Africa (6%), and others (15%) [19].

The final disposal of CS creates environmental problems worldwide. There is a need to investigate alternative uses for CS as a raw material for other processes. The chemical properties of CS, with it being rich in iron oxides, are unique and have attracted attention to the production of high-value-added materials for environmental applications to be used as sorbents, catalysts, or as a source of reactive species in ecological engineering [20]. Several applications for CSs have been found in the last three decades, mainly as a low-cost additive in construction materials [6]. The proposal to use CS as a mining photocatalyst represents a promising avenue as a semiconductor to be used as a photocatalytic material for water treatment and H_2_ production.

**Figure 1:**
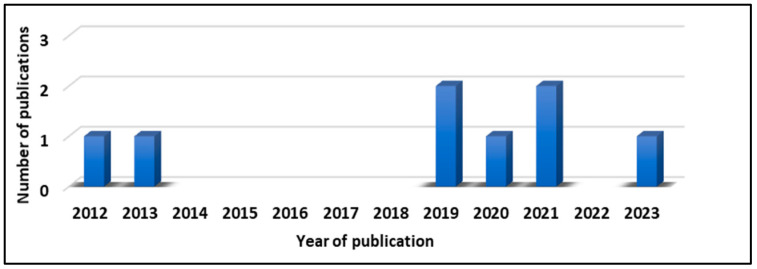
Publication treating photocatalysis and CS and contaminant treatment and H_2_ production.

Since 2012, just eight studies have been published exploring CS as a photocatalytic material. Figure 1 shows the number of publications using CS in the degradation of organic pollutants, such as industrial dyes, volatile organic compounds, and polycyclic aromatic hydrocarbons [10,11,20,21,22,23,24,25]. However, only two indicate H_2_ production using photocatalysis-assisted solar electrolysis [11,23].

This review provides a unique exploration of the largely untapped potential of copper slag as an affordable and sustainable photocatalyst for hydrogen production and water treatment. Currently, copper slag is primarily used in construction, where its commercial value remains low; however, its application as a photocatalyst could significantly enhance its market value. In contrast to previous studies focused on conventional photocatalysts, this work assesses the photocatalytic properties of copper slag, seeking to bridge the gap between waste valorisation and clean energy generation. By addressing both the environmental benefits and technological promise of copper slag, this review establishes a novel foundation for advancing research in sustainable photocatalysis.

## 2. Physical and Chemical Properties of Copper Slag

CS is a residue produced in a pyrometallurgical process to obtain copper concentrated from a concentrate of sulfide minerals, which contains materials such as FeO, SiO_2_, Al_2_O_3_, CaO, and Cu [6]. In the pyrometallurgical stage, known as smelting, very high-temperature furnaces are used. Because of the high temperatures, immiscible liquid phases are produced in this process: the copper-rich matte (sulfide) and the CS called oxide [26]. Generally, CS is composed of 30–40% FeO/Fe_3_O_4_, 35–40% SiO_2_, 0–10% Al_2_O_3_, and 0–10% CaO, which vary in their presence in the slag due to (i) the nature of the ores, (ii) the nature of the fluxes, (iii) the nature of the concentrates, (iv) the operating conditions, as well as other factors related to the production process, see Appendix A, [27]. In terms of appearance and properties, naturally cooled CSs have a black colour and a glassy surface and are dense, lumpy, hard, and brittle (see Table 1). However, depending on the cooling pattern, which can be natural or water-quenched, they can differ in appearance, density, and shape (see Table 2) [18].

## 3. Mechanisms of Water Treatment and H_2_ Production by Photocatalytic Process

Solar H_2_ generation is among the most efficient methods for converting photons into electricity, chemicals, and heat, facilitated by the synergy of various solar materials, electrochemical devices, and photocatalyst processes [40]. In the same way, Fujishima et al. indicate that the electrochemical decomposition of H_2_O into H_2_ and O_2_ requires a voltage of 1.23 V between the anode and cathode when using solar panels (a wavelength of about 1000 nm of solar radiation). In the visible light spectrum, this energy is very effective in an electrochemical system to facilitate the decomposition of H_2_O. Fox et al. propose that producing solar H_2_ by photocatalysis uses semiconductor materials [41]. Photocatalysis is based on the principle that when a semiconductor is exposed to a light source with the appropriate wavelength, electrons from the valance band pass to the conduction band, leaving positive holes in the valence band [42]. The principle underlying photocatalysis is presented by Zhang et al.; Equations (1)–(3) describe the photocatalytic mechanisms of slag, composed mainly of oxides, such as Fe_2_O_3_ and rutile TiO_2_, which are activated by alkali and cause the separation of the generated electron–hole pairs that promote the formation of H_2_. On the other hand, Equation (4) shows the action of the sacrificial agent SO_3_^2^ trapping the photogenerated hole in the aqueous solution that favours the generation of SO_4_^2−^ and H^+^ [43].

(1) $${\mathrm{Fe}}_{2}O_{3}\;\overset{hv}{\to }\;{\mathrm{Fe}}_{2}O_{3}\;(h^{+})+e^{-}$$

(2) $${\mathrm{TiO}}_{2}\;\overset{hv}{\to }\;{\mathrm{TiO}}_{2}\;(h^{+})+e^{-}$$

(3) $$H_{2}O+e^{-}\to \;½\;H_{2}+{OH}^{-}$$

(4) $$½\;H_{2}O+\;½\;SO_{3}^{2-}\to \;½\;{SO}_{4}^{2-}+H^{+}$$

Morales-Perez et al. propose a Langmuir–Hinshelwood type mechanism with a single dual site type, in which water molecules and an acidic solution (AC) are adsorbed over the CS surface (Equations (5) and (6)); in turn, when the CS is irradiated (*h*υ), it generates the charge pair, the hole in the valence band (VB), and the electron in the conduction band (CB) (Equation (7)). The adsorbed water interacting with the hole generates the hydroxyl radical and an adsorbed proton (Equation (8)). On the other hand, the degradation of an acid solution (acting as a sacrificial agent) occurs using the produced hydroxyl radicals or the photogenerated holes, producing other acids (AC_n_), methane, and CO_2_ as the main products (Equation (9)). The desorption of the products regenerates the surface sites. H_2_ is produced by proton reaction using photogenerated electrons in the conduction band of CS (Equations (10) and (11)) [23]. Montoya-Bautista et al. interpreted the sacrificial agent as a scavenger, promoting the reduction in H_2_O by reacting with the h⁺ species generated on the CS surface, thereby preventing the recombination of the e^−^/h⁺ pair. The representation is as follows:

(5) $$H_{2}O+\mathrm{CS}\to H_{2}O_{\mathrm{ads}}$$

(6) $$(\mathrm{AC})•\mathrm{OH}+\mathrm{CS}\to (\mathrm{AC})•{\mathrm{OH}}_{\mathrm{ads}}$$

(7) $$\mathrm{CS}+hv\to h^{+}\mathrm{VB}+e^{-}\mathrm{CB}$$

(8) $$H_{2}O_{\mathrm{ads}}+h^{+}\mathrm{VB}\to •{\mathrm{OH}}_{\mathrm{ads}}+H_{ads}^{+}$$

(9) $$(\mathrm{AC})•{\mathrm{OH}}_{\mathrm{ads}}+•{\mathrm{OH}}_{\mathrm{ads}}\;(ó\;{\;h}^{+}\mathrm{VB})\to ({\mathrm{AC}}_{n})•\mathrm{OH}+{\mathrm{CH}}_{4}(g)+{\mathrm{CO}}_{2}(g)$$

(10) $$2H_{ads}^{+}+2e^{-}\mathrm{CB}\to H_{\mathrm{2ads}}$$

(11) $$H_{2\mathrm{ads}}\to H_{2(g)}+\mathrm{CS}$$

(12) $$H_{2}O+h+\to {\mathrm{HO}}^{•}+H^{+}$$

(13) $$2H^{+}+2e^{-}\to H_{2}$$

The hydroxyl radicals produced in Equation (12) are captured during the degradation of the sacrificial agent. Hydrogen (H_2_) production is linked to the number of electron–hole pairs generated during irradiation. Additionally, Equation (13) represents CS as an n-type semiconductor. Under these conditions, CS can become depleted due to the adsorption of H⁺ on the negatively charged catalyst surface [44]. The low H_2_ production is limited because the electrons are dispersed over a large area, and the H⁺ ions remain attached to the catalyst surface after the loss of hydroxyl groups. Solis-López et al. note that magnetite is a more efficient catalyst than hematite for heterogeneous Fenton reactions because magnetite contains a combination of Fe^2^⁺ and Fe^3^⁺ oxidation states. This combination enhances the rate of OH^−^ production, as the reaction rate of H_2_O_2_ with Fe^2^⁺ sites is significantly higher than with Fe^3^⁺ sites [20]. Garcia-Estrada et al. evaluated the catalytic ability of metallurgical CS for pesticide removal in real wastewater by the heterogeneous Photo-Fenton process at a circumneutral pH under natural solar irradiation (NSI) [22]. The following reactions indicate that the active sites (Fe^3+^ OH^−^) adsorb H_2_O_2_ over the iron oxide surface and form a complex (Equations (14)–(16)):

(14) $${Fe}^{3+}+{OH}^{-}+H_{2}O_{2}↔{\left(H_{2}O_{2}\right)}_{s}$$

(15) $${\left(H_{2}O_{2}\right)}_{s}↔{Fe}^{2+}{HO}^{2-}+H_{2}O$$

(16) $${Fe}^{2+}{HO}^{2-}↔{Fe}^{2+}+{HO}_{2}^{•}$$

Subsequently, Fe^2+^ catalyses the decomposition of H_2_O_2_ into ${HO}^{•}$ radicals, according to Equation (17):

(17) $$H_{2}O_{2}+{Fe}^{2+}↔{Fe}^{3+}{OH}^{-}+{HO}^{•}$$

At the same time, the oxidation of Fe^2+^ to Fe^3+^ occurs in the presence of oxygen radicals, according to Equations (18) and (19):

(18) $${Fe}^{2+}+O_{2}↔{Fe}^{3+}{OH}^{-}+{HO}_{2}^{•}$$

(19) $${Fe}^{3+}{OH}^{-}+{HO}_{2}^{•}/O_{2}^{-}↔{Fe}^{2+}+H_{2}O/{OH}^{-}+O_{2}$$

They demonstrated that the CS-H_2_O_2_-NSI system produced ${HO}^{•}$ [22].

According to Yadav et al., 2020 [45], in this mechanism, upon illumination, electrons in the valence band of CuO are excited by the conduction band, leaving behind holes that can participate in oxidation reactions. The rGO in the composite captures these excited electrons, facilitating their transfer to reduce H⁺ ions, thus increasing the hydrogen production rate. For the other way, the mechanism involves light-induced electron excitation within CeO_2_, followed by the transfer of these electrons to MoS_2_, where they reduce H⁺ ions to produce H_2_. This transfer reduces the electron–hole recombination rate within CeO_2_. Additionally, MoS_2_ provides active sites that contribute to the enhanced hydrogen production observed in the CeO_2_/MoS_2_ composite. The study highlights the quantum confinement effect in MoS_2_, which plays a role in its high photocatalytic efficiency [45,46].

## 4. Copper Slag as Electrode Material

The CS exhibits distinctive chemical attributes, particularly its valuable photocatalytic properties by the solar-driven oxidation of organic pollutants in industrial wastewater and H_2_ production. Montoya et al., in their investigations, indicated that the use of CS as a Fenton-type photocatalyst is both an innovative alternative for the reuse of these wastes and an environmentally friendly option. Also, it indicates that the studied CS material acts as an n-type semiconductor with a bandgap of 2.75 eV, with absorption energy mainly in the violet-green range of the visible spectrum [11]. Augustynski et al. discussed the principal factors that affect the selection of semiconductor materials for solar H_2_ production and water treatment: (i) bandgap energy, (ii) photo-corrosion stability, and (iii) flat band potentials [40,47]. The band gap is a crucial parameter for identifying the wavelength of light that facilitates the transfer of electrons. Table 3 presents the band gap energies and wavelength of semiconductors used in photocatalysis processes.

Zhang et al. studied a nanomaterial based on alkali-activated blast furnace slag as a new material for H_2_ production. They concluded that this slag-based synthesised nanomaterial has a mesoporous structure with the following key characteristics: (i) a large specific surface area, (ii) uniform pores, (iii) a high ion-interaction capacity, and (iv) the relative ease of synthesis [43]. Montoya-Bautista et al. demonstrated the band edges of fayalite and magnetite within CS [11]. It was observed that the conduction bands of fayalite are positioned above the redox potential required for H_2_ production [44]. The photogenerated holes in the valence band of fayalite show a high capacity for alcohol degradation. This is due to the valence band’s potential of fayalite being lower than the redox oxidation potential of alcohol, thus meeting the essential thermodynamic criteria for the simultaneous reduction of water components and oxidation of alcohols.

**Figure 2:**
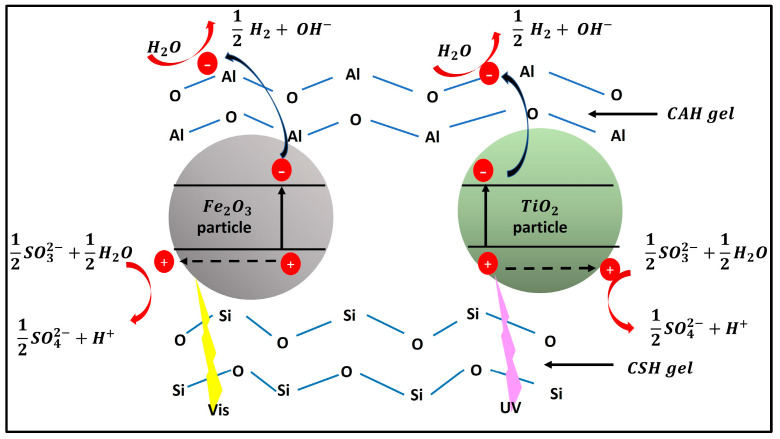
Schematic representation of the production of H_2_ on the granulated blast furnace slag catalyst activated with alkali by photocatalytic decomposition of water. Adapted from Ref. [43]. Copyright 2014.

Arzate-Salgado et al. evaluated the effectiveness of CS as a Fenton-type photocatalyst. They found that CS, when in an acidic medium with the presence of H_2_O_2_ and exposed to artificial sunlight, successfully degraded diclofenac dissolved in an aqueous solution [54]. Similarly, Huanosta-Gutiérrez et al. and Solís-López et al. demonstrated that CS also exhibits high photocatalytic activity in the Photo-Fenton process, both in an acidic medium combined with H_2_O_2_ and under UV radiation or artificial sunlight [10,20]. As a result, this alkali-activated blast furnace slag-based nanomaterial emerges as a highly efficient H_2_ producer due to the synergistic interaction between its oxide components and alkaline properties [43]. Figure 2 illustrates the H_2_ production mechanism in the alkali-activated granulated blast furnace slag catalyst through photocatalytic water-splitting [53,55,56,57].

Guo et al. indicate that α-Fe_2_O_3_ is a promising material for photocatalytic applications due to its narrow band gap of about 2.1 eV, chemical stability, and non-toxicity, which absorbs light up to 600 nm, collects up to 40% of the energy in the solar spectrum, and could be one of the cheapest semiconductor materials. In general, sunlight can be divided into three compositions according to wavelength: ultraviolet light (UV, k < 400 nm, E > 3.20 eV, accounts for about 4% of all solar energy), visible light (vis, 400 nm < k < 800 nm, 3.20 > E > 1.60 eV, accounts for about 43%), and near-infrared light (NIR, k > 800 nm, E < 1.60 eV, accounts for about 53%) [12,58]. Zhang et al. investigated a cementitious material comprised of granulated blast furnace slag activated with alkali, which catalysed the photocatalytic decomposition of water to synthesise H_2_ [43]. Mercado-Borrayo et al. indicate that the band gap of fayalite, the majority phase of CS, is 2.5 +/− 0.1 eV, making it capable of absorbing the blue-green part of the visible spectrum. This allows sunlight to be used as an energy source for photocatalysis instead of relying primarily on UV radiation [59]. García-Estrada et al. demonstrate the high efficiency and stability of metallurgical CS as a heterogeneous Fenton-type catalyst for the degradation of trace thiabendazole in different contaminated aqueous matrices, using the CS-H_2_O_2_ system at circumneutral pH under natural solar irradiation (NSI) [22,24]. Takata et al., in their latest research, aim to develop photocatalysts that can utilise a larger portion of the solar spectrum (longer wavelength) and can stably evolve H_2_ and O_2_ gases over several days [50].

**Figure 3:**
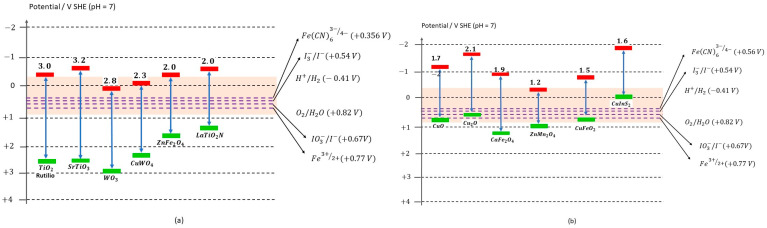
Schematic diagram of some semiconductors (**a**) type n and (**b**) type p. Adapted from Ref. [62].

Lasia et al. explain that in the presence of a conductive electrode, either metallic or glassy carbon, in contact with an electrolytic solution, an excess of electronic charge accumulates on the electrode surface, resulting in a charge distribution mainly within the solution. In the case of semiconductor electrodes, the concentration of conductive species (electrons or holes) is significantly lower than that of the surrounding solution. Consequently, a redistribution of space charge occurs within the semiconductor electrode. Depending on how the charge is distributed within the conduction and valence bands, a potential bending occurs at the surface. In the case of an open-circuited n-type semiconductor electrode, this bending is directed upwards from the band edges. In contrast, p-type semiconductors exhibit a bending downward from the band edges [60,61]. Montoya-Bautista et al. evaluated CS using electrochemical techniques to measure its properties as photocatalyst material and demonstrated the electrochemical and photoelectrochemical behaviour using the Mott–Schottky model. Five different values of frequency (100–1000 Hz) were used to detect the presence of semiconductors in CS, distinguishing (i) fayalite and (ii) magnetite, respectively. The lower part of the conduction band (CB) must have a potentially more negative value than the reduction of H^+^ to H_2_ (0.41 V v/s NHE). Likewise, the upper part of the valence band (VB) must exceed the oxidation potential of H_2_O to O_2_ (0.82 V v/s NHE). Therefore, the bandgap energy (Eg) of the photocatalysts must be at least 1.23 eV to achieve water-splitting [57]. Figure 3 represents a schematic band diagram of the photocatalytic performance of semiconductors (a) type n and (b) type p. The orange horizontal range indicates the redox potentials of OER and HER, and the purple dashed horizontal lines indicate the redox potential, respectively [62,63,64].

**Figure 4:**
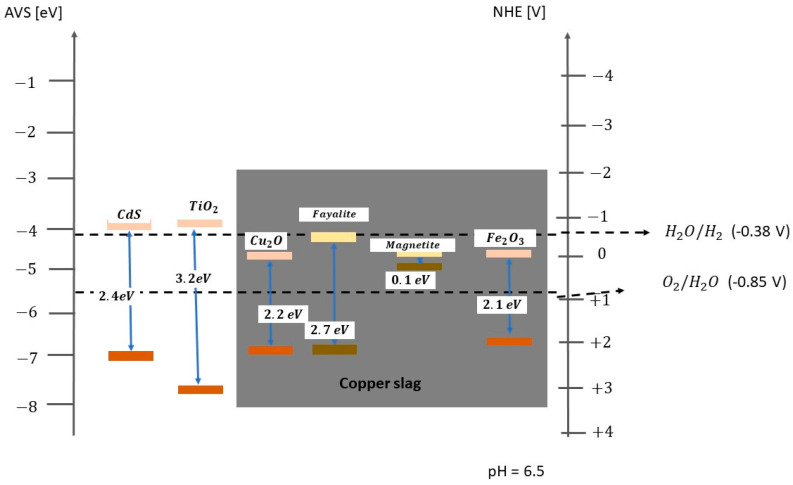
Bandgap diagram for CS and other semiconductors at pH = 6.5. The low bands, in a dark colour, are the valence bands (VB), and the high bands, in light colour, are the conduction bands (CB), and in the centre the band gap values. Mineralisation is possible if the potential is within the band gap (AVS: absolute vacuum standard) [11]. Copyright 2020.

Figure 4 shows that the conduction bands of fayalite are above the redox potential for H_2_ production [62]. The photogenerated holes residing in the valence band of fayalite exhibit the ability to degrade alcohol efficiently. This ability stems from the fact that the valence band potential of the fayalite falls below the redox oxidation potential of the alcohol, thus satisfying the essential thermodynamic criteria for the concurrent reduction of water constituents and oxidation of alcohols [11,65].

Montoya-Bautista et al. used CS as a photocatalytic material. The test was performed using different electrolytes such as methanol, isoamyl alcohol, and propanol. The main objective was to evaluate the efficiency of these compounds in the generation of H_2_ by a photocatalytic process. H_2_ production showed higher rates when subjected to simulated solar irradiation compared to mercury irradiation. These results highlight the importance of both the type of compound and the light source in influencing H_2_ production by photocatalysis [11]. The use of industrial by-products or waste materials as photocatalysts has emerged as a promising approach to curb the production costs associated with titanium semiconductor-based materials. Among these by-products, crude or modified metallurgical slags are a viable option due to their cost-effectiveness, wide availability, and favourable optical properties.

Alternative H_2_ production methods such as (i) steam reforming, (ii) electrolysis, (iii) thermolysis, (iv) photobiolysis, and (v) photolysis, among others, can be classified according to the electrolyte used. There is great interest in photochemical processes such as (i) photoelectrolysis and (ii) photocatalysis. Lin et al. recommend using photocatalysis because it only requires a light source, water, and a semiconductor powder or photocatalyst [66,67,68,69,70,71,72].

Comparing the various photocatalytic materials shown in Table 4 reveals unique characteristics regarding their performance in water treatment and solar H_2_ production applications. TiO_2_ stands out as the most studied photocatalytic material; it was first used for water photolysis, demonstrating its effectiveness in H_2_ production; however, its reliance on UV light limits its application under direct sunlight, which has driven the development of alternative materials capable of absorbing visible light. In contrast, CS has shown promising results in the degradation of organic compounds like phenol and toxic alcohols. Although it does not reach the same efficiency level as TiO_2_ in hydrogen production, its low cost and dual function—(i) contaminant oxidation and (ii) H_2_ generation—make it an affordable option for photocatalysis, especially in processes that utilise solar irradiation. This feature is crucial for large-scale applications where costs are a significant limitation, and waste recycling is a sustainability goal for the mining industry. Materials like g-C_3_N_4_ with TiO_2_ show that nanoscale modifications can considerably improve photocatalytic efficiency. Nanotubes decorated with silver nanoparticles increase H_2_ production by enhancing the charge transfer under UV and visible light, partially resolving the electron–hole recombination issue in pure TiO_2_. For its part, g-C_3_N_4_ exhibits superior visible light absorption, making it ideal for sustainable solar applications. Another notable material is the TiO_2_/SiO_2_ nanocomposite, which combines the stability of SiO_2_ with the photocatalytic efficiency of TiO_2_, achieving high effectiveness in organic pollutant degradation and H_2_ production simultaneously.

The combination of materials, such as the ZnO/TiO_2_/g-C_3_N_4_ composite and the heterogeneous Photo-Fenton process with CS, highlights the trend towards using materials that enhance efficiency and reduce costs. The ZnO/TiO_2_/g-C_3_N_4_ composite stands out for its capacity to increase solar H_2_ production efficiency and pollutant degradation by combining the properties of each component, achieving improved visible light absorption and greater photocatalytic activity. Similarly, the Photo-Fenton process using CS under solar irradiation enables the reuse of industrial wastewater, such as from the textile industry, by removing harmful contaminants and residual H_2_O_2_, representing an economical and sustainable alternative. This advanced oxidation process utilises Fe-based reagents present in CS, which, combined with oxidants like NaOCl under simulated sunlight, has demonstrated improved pollutant degradation rates. The CuO/rGO composite benefits from rGO’s ability to act as an electron acceptor, which enhances charge separation by reducing the recombination rate of photogenerated electron–hole pairs and CeO_2_/MoS_2_ MoS_2_, provides active sites that contribute to the enhanced hydrogen production observed in the composite. The study highlights the quantum confinement effect in MoS_2_, which plays a role in its high photocatalytic efficiency.

Table 5 presents the methods of materials used to produce H_2_ through the photocatalysis process.

Table 5 provides an analysis of H_2_ production and contaminant degradation percentages using different alcohols such as methanol, propanol, and isoamyl alcohol as reactants under two types of lamps: Mercury (Hg) and Xenon (Xe). The main parameters considered are the average H_2_ production rate (in µmol g^−1^ h^−1^) and the apparent quantum yield (AQY), along with the degradation efficiency (%) of the alcohol substrates. Under the Hg lamp, methanol achieves the highest average H_2_ production rate at 0.519 µmol g^−1^ h^−1^, followed by propanol at 0.414 µmol g^−1^ h^−1^, and isoamyl alcohol with the lowest rate at 0.221 µmol g^−1^ h^−1^. When using the Xe lamp, H_2_ production rates and AQY values increase for all alcohols. Methanol again achieves the highest H_2_ production at 0.867 µmol g^−1^ h^−1^, followed by propanol at 0.649 µmol g^−1^ h^−1^ and isoamyl alcohol at 0.588 µmol g^−1^ h^−1^. In both lamp configurations, methanol consistently produces the highest H_2_ values, making it the most efficient alcohol under photocatalytic conditions. * The Degradation reported after 6 h of reaction [25,43,57].

## 5. Photocatalytic Reactors Are Used for H_2_ Production and Water Treatment

Focusing on the prospects of a green H_2_ economy, a photocatalytic device is proposed that uses solar energy for water-splitting and produces H_2_. Since both reactions require significant amounts of kinetic overpotential, a practical photocatalyst would need to have a band gap energy in the range of 1.6–2.4 eV to drive overall water-splitting [78].

**Figure 5:**
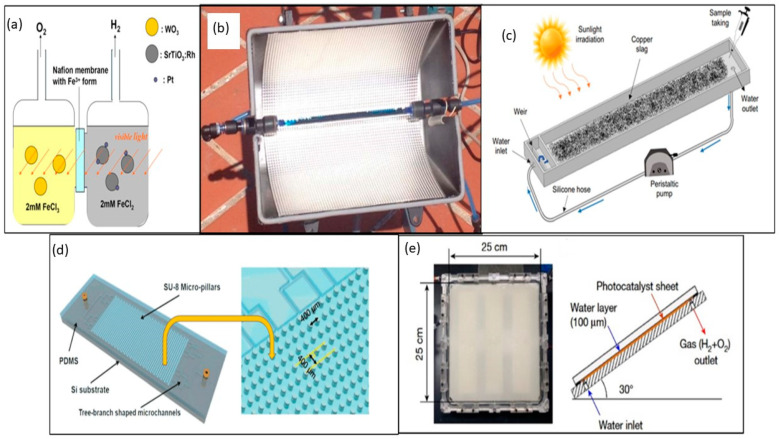
(**a**) Schematic diagram of the dual-chamber reactor system [83]. (**b**) Photocatalytic cell fixed bed packing, Reprinted from Ref. [80]. (**c**) Heterogeneous photocatalytic treatment of water can be bifunctional if H_2_ is produced during the degradation of pollutants [24]. (**d**) Schematic of the high surface area optofluidic microreactor with micro-pillar structure and cross section of the staggered micro-pillars in the reaction chamber [81]. (**e**) panel reactor unit and the structure of the panel reactor unit [82]. Copyright 2021.

In 2012, Dincer et al. comprehensively outlined sustainable and environmentally friendly methods for H_2_ production, classifying them according to their driving sources and applications shown in Figure 5a [79]. Borges et al. conducted a study on solar photocatalysis using a continuous fixed-bed reactor configuration. This configuration comprises a glass cylinder placed in the core of a parabolic solar collector, inside which the photocatalytic material is packed—in this case, volcanic sand serves as the photocatalytic material. This photoreactor achieves a greater contact between the photocatalyst and the dissolution of pollutants, resulting in more scalable equipment from an industrial point of view, as depicted in Figure 5b [80]. Herrera-Ibarra et al. investigated, for the first time, the application of CS in the treatment of wastewater from a textile industry using the solar heterogeneous solar Photo-Fenton process (due to the use of Fe material) under solar irradiation (see Figure 5c). For the experiments, the first thing they did was adjust the pH of the wastewater by acidifying it; then, they placed the reactor under natural sunlight, and the CS was deposited on the bottom of the reactors, distributing it evenly. Wastewater was added to each reactor and 30% H_2_O_2_. Finally, the water mixture was recirculated at a constant flow rate. CS and exposure to sunlight did not contribute to water toxicity. Li et al. developed an optofluidic microreactor with staggered micropillars in the microreaction chamber (see Figure 5d). They enlarged the surface area for loading the photocatalyst and induced turbulence in the fluid flow, increasing the mass transfer to boost the H_2_ production rate [81]. Kim et al. propose a photocatalytic water-splitting system [78], as shown in Figure 5e. The photocatalytic reactor comprises a UV-transparent glass window and a photocatalyst sheet (SrTiO_2_/Al). The polyurethane pipe is used to transport gaseous products and reactive water [82]. The photocatalytic reactors play a crucial role in solar H_2_ production, enabling efficient solar energy conversion into clean, sustainable, and storable H_2_. The use of CS as a photocatalytic semiconductor will help reduce the demand for pure materials, reduce the need for new extraction processes, and reduce the amount of waste generated in the mining industry that is dispersed around the world.

## 6. Discussion

The number of published manuscripts is limited, and they show significant variations in their experimental methodologies as well as in the photoreactors designed and used in the production of solar H_2_ and the treatment of contaminated waters. Therefore, creating a review manuscript that gathers all the information related to the production of solar H_2_ using photocatalytic processes and CS will help to standardise experimental methodologies for the future. The direct use of CS as a photocatalytic material for solar H_2_ production is an interesting and promising research topic; however, in recent years, it has gained greater importance due to the need to produce energy cheaply, sustainably, cleanly and by applying concepts of a circular economy in mining. The use of CS as a photocatalyst for H_2_ production presents a significant opportunity both for the recovery of mining waste and for the generation of sustainable energy. With the significant volume of slag generated by the mining industry, one of its main advantages lies in its abundant availability. Reusing this material can reduce the accumulation of environmental liabilities and optimise resource efficiency. The photocatalytic performance of CS can be attributed to its chemical composition and distinctive surface properties. Its broad surface area enhances the adsorption of reagents and catalytic activity. Moreover, the presence of metal oxides such as iron oxide and copper oxide in the slag acts as active sites to promote photocatalytic reactions. The use of nanometric CS material allows photocatalytic activity to change from UV wavelengths to sunlight, transforming this waste into a semiconductor, thus achieving industrial massification that can be used in water treatment and H_2_ production by photocatalysis. However, this field of research presents many technical challenges that slow the development of such technologies. The most important considerations are (i) the optimisation of CS photocatalytic properties, (ii) an analysis of the actual bandgap, and (iii) surface modifications to improve its catalytic activity and stability over time. This would notably aid in the development of more efficient photocatalytic reactors to maximise H_2_ production rates. The presence of metal oxides such as iron oxide and copper oxide in the slag serves as active sites to promote photocatalytic reactions. Therefore, reusing CS as a photocatalyst is an opportunity to contribute to environmental sustainability.

Regarding other studies where slags are used, it can be argued that there are different results and perspectives; however, it is possible to gather interesting and useful information. Yao Jun Zhang et al. (2014) [43] used nanometric materials based on Fe_2_O_3_ and TiO_2_ as catalysts in the production of H_2_ through water photolysis. Both materials exhibit mesoporous structures that act as active species, favouring H_2_ evolution and generating 52 µmol/g in 6 h of operation under artificial irradiation, which is the only recorded data regarding H_2_ production. Additionally, the same author in 2017 continued using alkali-doped slags, optimising the photocatalytic process and efficiency in H_2_ production through the refinement of material synthesis and characterisation, achieving up to 2.4 times more production compared to using non-alkali-activated slag. These studies are pioneering in the valorisation of industrial waste with clean energy production. However, Le Kang et al. (2017) [84], Montoya et al. (2018) [44], Montoya et al. (2019) [25], and Yao Jun Zhang et al. (2020) [85] work on advanced techniques for alkali activation and the synthesis of new materials, which were studied under controlled irradiation conditions but do not specify the amount of H_2_ produced, only indicating that efficiency improvements in production have been made, respectively.

The mesoporous structure of slag is a result of alkali activation. This structure promotes the H_2_ evolution reaction (HER) because it is better able to separate photogenerated charges. Consequently, alkali activation and irradiation conditions are understood to be fundamental parameters for optimising photocatalytic H_2_ production using slags. The main disadvantages of photocatalytic reactors and their materials are understood to be (i) low quantum efficiency, as only a fraction of the incident photons will be converted into electrons transferred to generate the H_2_ evolution reaction; (ii) low light absorption, as slags have very limited UV absorption ranges, reducing the system’s efficiency; (iii) recombination of electron–hole pairs, a common problem in photocatalytic reactors, which reduces the number of electrons available for water reduction, thereby lowering the efficiency of the H_2_ evolution reaction; (iv) some results indicate that slags may degrade over time, reducing the electrochemical device’s lifespan; (v) limitations in mass transport of reactants and products to and from the slag surface, controlling the kinetics of H_2_ production.

Regarding photocatalytic reactors published in the scientific literature, it has been indicated that the use of metal oxides based on slag leads to the recombination of electron–hole pairs, thereby reducing the efficiency of photocatalytic mechanisms. On the other hand, the use of curved glass and the distance between the light emission could increase light dispersion, reducing direct light incidence. Additionally, an increase in internal pressure and temperature control has been observed, which affects the stability of the slag photocatalyst and the repeatability of the results. The use of stainless-steel reactors significantly improves the handling of overpressure and high temperatures. The use of flat Pyrex glass significantly aids in the uniform distribution of light, making this design suitable for small-scale photocatalytic studies. The use of a quartz plate improves radiation absorption; however, it does not improve the homogeneous distribution of light.

Under certain conditions, filamentous carbon formation was observed on the slag during prolonged reactions at elevated temperatures, which would eventually reduce the active sites of the mesoporous material, lowering the efficiency of the H_2_ evolution mechanism. Finally, photoelectrochemical systems using slag as a photocatalyst present issues with photoactivity due to their varied composition and impurities in the material matrix, which could reduce H_2_ production over time. On the other hand, large quantities of slag may be needed, which would help environmental decontamination. The water and gas feed flow are not parameters studied in detail by the authors.

The limitations of using CS as a photocatalytic material are high, and it is necessary to study the individual characteristics of the components, as it is not yet known which element produces H_2_ in greater quantity within its multicomponent composition of CS. Moreover, it is imperative to assess the environmental footprint and potential toxicity associated with the use of CS as a photocatalyst in contact with water. The direct utilisation of copper slag as a photocatalytic material for H_2_ production via solar energy holds significant promise in the realm of renewable energy. Nonetheless, additional research and development efforts are warranted to fully explore the extent of its functionality and applicability. These efforts, in turn, will make a substantial contribution towards shaping a cleaner and more sustainable energy landscape for the future.

The use of industrial by-products or waste materials as photocatalysts has emerged as a promising approach to curb the production costs associated with titanium semiconductor-based materials. Among these by-products, crude or modified metallurgical slags are a viable option due to their cost-effectiveness, wide availability, and favourable optical properties. Numerous investigations have shown that both steel and blast furnace slag can remove chemical contaminants, degrading dyes and even produce H_2_ under simulated solar Irradiation [25,43,57]. Because the other components of the slag could interfere with the photocatalytic mechanism of CS, it is necessary to know the photocatalytic properties of solar photocatalysis of CS, including bandgap engineering and surface modification, is essential to improve its catalytic activity and stability.

There are no economic studies on the use of copper slag in the process of water treatment. Research shows that it is feasible to use in the process for water treatment and hydrogen production. Phiri et al. analysed the potential benefits of reprocessing copper slag waste using the circular economy model. It shows that the application of circular economy principles to copper slag waste has the potential to create additional economic value, improve the energy efficiency of metal production, increase the supply of critical metals and reduce the impact of metal production on the environment [86].

## 7. Current Challenges and Future Perspectives

Historically, CS has been primarily used in construction as part of concrete or asphalt, where its chemical and electrochemical properties are not fully utilised. However, there has been growing interest in the potential of CS for high-value environmental applications, with emerging uses in fields such as catalysis, adsorption, and the generation of reactive species for contaminant degradation. The valorisation of CS for water treatment and solar H_2_ production offers an innovative approach that tackles the challenges of mining waste management and promotes industrial sustainability. CS has traditionally been seen as a low-value waste, mainly destined for authorised slag heaps or used in construction materials. However, leveraging its chemical and physical properties opens new potential applications with significant environmental and economic benefits. The use of CS in water treatment can help reduce the volume of CS requiring disposal, ease pressure on authorised disposal sites, avoid the creation of illegal dumping grounds, and thus lower environmental contamination risks associated with improper waste management. Furthermore, it provides a practical solution for the metallurgical industry to repurpose its by-products. Significant economic savings can also be achieved by capitalising on the inherent physical, chemical, and electrochemical properties of CS. Its use in low-cost electrochemical devices for water treatment can reduce reliance on costly chemical reagents essential for water treatment, especially in financially constrained companies. This cost-saving potential is crucial for industries needing sustainable and affordable solutions for pollutant removal. Additionally, the use of CS in water treatment extends access to water resources by enabling the treatment of contaminated sources that would otherwise be unsuitable for human consumption or agricultural irrigation, which is particularly vital in arid and hyper-arid regions like the Atacama Desert in Chile. Beyond water treatment, CS also shows strong potential as a photocatalyst for solar H_2_ production, offering an economical and efficient pathway for producing clean, solar-derived H_2_. Producing H_2_ through photocatalysis using CS is an emerging field, as solar-driven reaction processes, such as the Photo-Fenton reaction, present a sustainable and clean alternative for H_2_ production while contributing to the mining industry’s circular economy by creating a new business. However, several challenges must be addressed to fully utilise the potential of CS. Optimising CS remains an area requiring careful attention; enhancing its photocatalytic properties through surface modifications is essential to improve charge transfer, thus increasing H_2_ production rates and making CS a more competitive photocatalyst compared to TiO_2_. This requires precise and robust engineering to tailor its surface properties and enhance efficiency under solar irradiation. Another major challenge is the design and scalability of reactors and electrodes; transitioning CS-based photocatalysis from laboratory devices to industrial-scale applications requires more efficient reactor and electrode designs that maximise light absorption and mass and charge transport, ensuring that H_2_ evolution reactions can proceed at high rates. In addition, advanced reactor configurations that enhance surface area, light distribution, and reactant flow will be essential for scaling to commercial levels. Reusing CS not only lowers costs but also reduces the carbon footprint by recycling mining waste, aligning with the goals of a circular economy. However, understanding the true economic impact of using CS in the long term and its potential market value as a photocatalyst material will require extensive research. Reflecting on the future prospects of CS, emerging technological devices play a crucial role in developing efficient and sustainable methods for both (i) water treatment and (ii) H_2_ production. Future research is likely to focus on areas such as thermochemical modifications and the creation of nanocomposites that incorporate metal oxides within CS. By integrating nanoscale oxides and using solar simulators to test photocatalytic efficiency, CS can be optimised as a sustainable and effective option for catalytic processes that use sunlight as an energy source.

## 8. Conclusions

The direct use of CS as a photocatalytic material for solar H_2_ production and water treatment in areas with high solar radiation stands out as a sustainable solution. Its use as a photocatalyst maximises resource efficiency and reduces waste. Optimising photocatalytic properties, designing reactors, and assessing environmental impacts are crucial aspects that must be addressed in future research. Precisely because of the presence of other elements in CS, there will be obstacles in the photocatalysis mechanism of CS during solar H_2_ production or water treatment. Studies show that CS is a chemically stable and efficient Fenton-type catalyst for the degradation of micro and macro contaminants present in wastewater. Overall, the literature review highlights the potential of using CS as a photocatalytic material for solar H_2_ production; however, deep research and development efforts are needed to optimise and design efficient systems. By exploring and overcoming these challenges, the direct use of CS can contribute to a more sustainable and cleaner energy future for the planet. This review manuscript systematically and in detail compiles the alternative use of CS as an electrocatalytic material, broadening the horizon for the use of this environmental liability and clearly improving its market price.

## Figures and Tables

**Table 1:** Physical and mechanical properties of CS [6,28].

Property	Value
Unit weight (T/m^3^)	2.8–3.8
Absorption	0.13
Bulk density (T/m^3^)	2.3–2.6
Conductivity (µs/cm)	500
Specific gravity	2.8–3.8
Hardness (Moh)	6–7
Moisture (ppm)	<5
Abrasion loss (%)	2.4–10
Internal friction angle	40–53

**Table 2:** Visual description of copper smelting slag.

Property	Description	Ref.
Particle shape	Angular	[29,30,31]
	Irregular	[32,33]
	Multifaceted	[34]
Surface Texture	Glassy	[30,31,33,35]
	Smooth	[18,31,34]
	Granular	[27,36,37]
	Rough	[30]
Color	Black	[23,30,31,33]
	Blackish grey	[34,38]
	Brown with green, red, or black tint	[35,39]

**Table 3:** The band gap of typical component of CS.

Photocatalyst Material	Band Gap	Wavelength	Ref.
	[eV]	(nm)	
TiO_2_ (anatase)	3.2	380	[48,49]
TiO_2_ (rutile)	3	414	[49]
Copper Slag (CS)	2.75	450	[11,50]
Fe_2_SiO_4_	2.7	---	[11,51]
Fe_2_O_3_	2.3	565	[51,52]
α-Fe_2_O_3_	1.9	600	[53]

**Table 4:** Literature related to water treatment and H_2_ production by photocatalysis.

Ref.	Photocatalytic Material	Photocatalytic Material	Key Results
[73]	TiO_2_	Water electrolysis with illuminated TiO_2_.	Production of hydrogen from water; The first study to document the photolysis of water.
[10]	Copper Slag	Photocatalytic degradation of phenol.	Degradation of phenol.
[43]	Alkali-activated blast furnace slag	Photocatalytic decomposition of water.	Decomposition of water.
[54]	Residuos metalúrgicos	Photodegradation of Diclofenac.	Fenton-type photocatalysts with potential applications in advanced oxidation processes (AOPs) assisted by sunlight at near neutral pH.
[57]	Au nanoparticles	Total photocatalytic splitting of water	Improved the efficiency of the hydrogen production rate relative to TiO_2_ nanoparticles
[59]	Metallurgical slag	Photocatalysis of water.	Applications in highly efficient advanced chemical oxidation processes.
[74]	Surface-modified titanate nanotubes	Spin trap under irradiation of photocatalysts prepared with ultraviolet or visible light.	Increased hydrogen production was observed on surface-modified titanate nanotubes by 5-amino acid salicylic acid decorated with nano-sized silver nanoparticles.
[75]	g-C_3_N_4_	Photocatalysis under controlled conditions	Significantly improve hydrogen production compared to conventional TiO_2_.
[76]	TiO_2_/SiO_2_ nanocomposites	Decomposition of organic pollutants and hydrogen production at the same time.	High photocatalytic efficiency for water treatment and H_2_ production.
[11]	Copper Slag	Photocatalytic degradation of toxic alcohols.	Efficient photocatalytic reaction for the oxidation of organic pollutants in industrial wastewater, with the simultaneous generation of H_2_.
[77]	ZnO/TiO_2_/g-C_3_N_4_	Roller experiments and product evaluation.	Increasing Hydrogen Production Efficiency and Pollutant Degradation.
[24]	Copper slag	Photo-Fenton (HPF) heterogeneous process under solar irradiation.	This water can be reused in the textile industry process if the time of exposure to the sun is increased to remove residual H_2_O_2_.
[22]	copper slag (CS) as an iron-based catalyst, and NaOCl as oxidants	Advanced Oxidation Processes using simulated sunlight (SSL).	It improved the rate of degradation of the pollutant, compared to the rate of the Photo-Fenton reaction.
[45]	CuO and CuO/rGO	Photocatalysts by hydrothermal route	The rGO in the composite captures these excited electrons, facilitating their transfer to reduce H⁺ ions, thus increasing the hydrogen production rate
[46]	CeO_2_/MoS_2_	MoS_2_ provides active sites that contribute to the enhanced H2 production observed in the CeO_2_/MoS_2_ composite	The CeO_2_/MoS_2_ composite is synthesised to leverage both materials’ photocatalytic properties under visible light.

**Table 5:** Hydrogen production by photocatalysis.

Lamp	Hg			Xe		
Alcohol	Average H_2_ Production (µmol g^−1^h^−1^)	AQY H_2_	Degradation * (%)	Average H_2_ Production (µmol g^−1^h^−1^)	AQY H_2_	Degradation * (%)
Methanol	0.519	0.01	17.32	0.867	0.22	13.94
Propanol	0.414	0.008	3.29	0.649	0.08	3.49
Isoamyl	0.221	0.004	3.42	0.588	0.01	1.98

* Degradation reported after 6 h of reaction.

**Figure A1:**
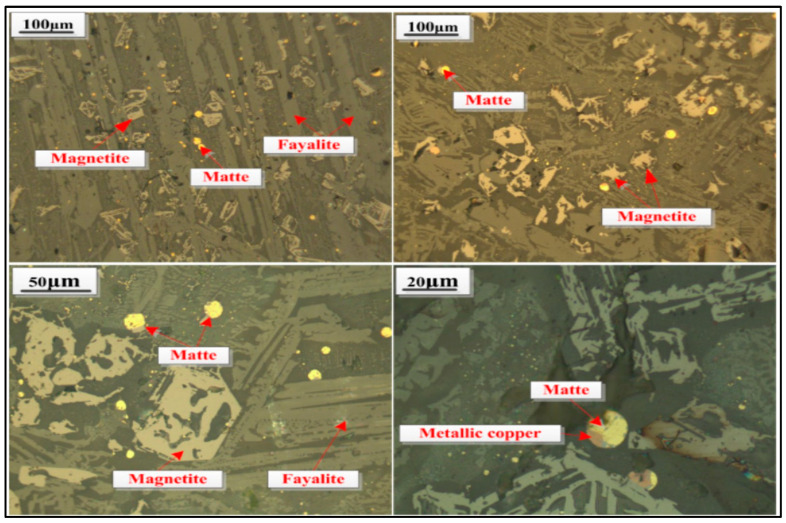
Microstructure of CS, Tongling, China. Reprinted from Ref. [91].

## Data Availability

No new data were created or analyzed in this study.

## Appendix A. Superficial Characterisation of CS

The chemical characterisation of CS allows for identifying the compounds present in them by X-ray fluorescence (XRF) [87]. The crystalline phases of CS are generally composed of fayalite (Fe_2_SiO_4_), magnetite (Fe_3_O_4_), quartz (SiO_2_), and some iron and copper sulfides. The predominant elements contained in CS are Fe and Si [88]. Phiri et al. delved into the feasibility of incorporating CS as a valuable resource within the framework of the circular economy. The researchers meticulously compiled data on the average chemical composition of these waste materials from a comprehensive review of 80 previous studies conducted in 21 countries. These include Chile, Canada, Australia, China, the Democratic Republic of Congo, Finland, Italy, Iran, Kazakhstan, Namibia, Mexico, Serbia, Poland, Singapore, South Africa, South Korea, Spain, Turkey, the United States and Zambia, (see Table A1).

Several established techniques can be used for the comprehensive characterisation of CS, such as (i) optical microscopy, (ii) scanning electron microscopy (SEM), (iii) electron microprobe analysis (EMPA), (iv) X-ray diffraction (XRD), (v) X-ray fluorescence spectroscopy (XRF), and (vi) Raman spectroscopy. These methods provide valuable information on the phase composition of the material and the spatial distribution of metallic elements in the different phases. The integration of all these analytical approaches allows a comprehensive characterisation of the CS, covering its chemical composition and mineralogical properties [6].

**Table A1:** Average chemical composition of copper smelting slags for major and minor elements in 21 countries [86,89]. Copyright 2021.

Components	SiO_2_	FeO	Fe_2_O_3_	CaO	Al_2_O_3_		MgO	K_2_O	S	MnO	TiO_2_	P_2_O_5_		Na_2_O
(wt%)	33.18	32.15	31.93	6.21	5.88		2.02	1.53	1.19	0.42	0.40	0.39		0.06
Elements	Ca	Al	Zn	Ag	Mg	Cu	Pb	Co	As	Ti	Ni	Mn	Sb	Mo
(wt%)	3.05	2.59	1.83	1.67	1.36	1.19	0.94	0.48	0.38	0.28	0.24	0.22	0.10	0.06

X-ray diffraction (XRD) analysis determines the crystalline phases present and, in general, allows identifying and obtaining the relative abundance, i.e., estimating the amorphous percentage of the sample. For example, Figure A1 shows the crystalline phases of fayalite (Fe_2_SiO_4_), and magnetite (Fe_3_O_4_) [89].

Scanning electron microscopy (SEM) is a valuable tool that provides structural information to evaluate very small surface topographic details of whole objects or powders, due to its higher resolution [90]. Figure A1 presents the micrographs of the CS from Tongling, China; it is observed that fayalite and magnetite are the main minerals in CS, and they are closely combined with copper matte and the glassy phase.

Many researchers use SEM and DRX images to identify the surfaces’ morphological characteristics, the material’s crystalline structure, and their chemical composition [13,14,24,25,28,31,35,37,44,55,59,85,86,91,92,93,94,95,96,97,98,99,100,101,102,103]. The presence of metal oxides such as iron oxide and copper oxide in the slag serves as active sites to promote photocatalytic reactions. Therefore, reusing CS as a photocatalyst is an opportunity to contribute to environmental sustainability and envisions an opportunity for H_2_ generation.
